# YOLOv5s-g^n^Conv: detecting personal protective equipment for workers at height

**DOI:** 10.3389/fpubh.2023.1225478

**Published:** 2023-09-28

**Authors:** Huihua Chen, Yaoyu Li, Huanxi Wen, Xiaodong Hu

**Affiliations:** School of Civil Engineering, Central South University, Changsha, Hunan Province, China

**Keywords:** falling from height, personal protective equipment, workers working at height datasets, image augmentation, deep learning, you only look once (YOLO), YOLOv5s-g^n^Conv

## Abstract

**Introduction:**

Falls from height (FFH) accidents can devastate families and individuals. Currently, the best way to prevent falls from heights is to wear personal protective equipment (PPE). However, traditional manual checking methods for safety hazards are inefficient and difficult to detect and eliminate potential risks.

**Methods:**

To better detect whether a person working at height is wearing PPE or not, this paper first applies field research and Python crawling techniques to create a dataset of people working at height, extends the dataset to 10,000 images through data enhancement (brightness, rotation, blurring, and Moica), and categorizes the dataset into a training set, a validation set, and a test set according to the ratio of 7:2:1. In this study, three improved YOLOv5s models are proposed for detecting PPE in construction sites with many open-air operations, complex construction scenarios, and frequent personnel changes. Among them, YOLOv5s-gnconv is wholly based on the convolutional structure, which achieves effective modeling of higher-order spatial interactions through gated convolution (gnConv) and cyclic design, improves the performance of the algorithm, and increases the expressiveness of the model while reducing the network parameters.

**Results:**

Experimental results show that YOLOv5s-gnconv outperforms the official model YOLOv5s by 5.01%, 4.72%, and 4.26% in precision, recall, and mAP_0.5, respectively. It better ensures the safety of workers working at height.

**Discussion:**

To deploy the YOLOv5s-gnConv model in a construction site environment and to effectively monitor and manage the safety of workers at height, we also discuss the impacts and potential limitations of lighting conditions, camera angles, and worker movement patterns.

## 1. Introduction

Working at height is one of the most hazardous types of work. It is recognized by the International Labour Organization (ILO) as one of the leading causes of injury and death in the workplace (1). According to the latest figures from The Bureau of Labor Statistics (BLS), there were nearly 900 deaths in a year due to falling from height, slips, and trips in the workplace (2). Besides, the latest figures from the United Kingdom Health and Safety Executive (HSE) report that falls from height are the leading cause of fatal accidents for workers (3). Similarly, Safe Work Australia (SWA) states that working at height accounts for 13% of all work fatalities between 2015 and 2019 (4). Correspondingly, according to the statistics released by the Government of China in 2017–2019, the proportion of falling from height in construction safety production accidents accounted for 52.41%, and the number of deaths was also as high as 46.93% (5).Therefore, construction safety has become a global concern. Detecting personal protective equipment for workers at height and safety management has been a top priority in the construction industry worldwide.

Falls from height (FFH) has attracted wide attention from scholars worldwide. According to research of Heinrich’s (6), accidents can be attributed to two main factors: unsafe workplace conditions and unsafe worker behavior (6). Therefore, successfully identifying these workplaces’ unsafe conditions and behaviors, i.e., hazard recognition, is the foundation of improving construction safety (7). Unfortunately, approximately 57% of hazards on construction sites remain unidentified (8). The top four types of fatal construction safety accidents demonstrated by Occupational Safety and Health Administration (OSHA) statistics directly relate to unsafe worker practices (9). Unsafe behavior of construction workers is a significant cause of construction safety accidents, especially the failure to wear PPE in a standardized way (10). Expensive sensors are used for PPE detection in traditional construction sites (11). Consequently, there is an urgent need for a more intelligent and more affordable method of detecting PPE for construction workers working at height.

With the rapid rise in computer computing power in recent years, neural networks have returned to the public eye; many scholars use Computer Vision (CV) (12) to reduce the rate of safety accidents in the construction industry. In Computer Vision, the target detection algorithm is the most commonly used. It can be classified into two types: Two-stage and One-stage. Girshick et al. (13) proposed the Region-Convolutional Neural Networks (R-CNN) model based on AlexNet’s (14) research in image extraction in 2014, which can be seen as a crucial opening for deep learning algorithms for object detection, as well as pioneering work for Two-stage target detection algorithms, commonly represented by the RCNN series [Fast-RCNN, Faster-RCNN (15), and Mask-RCNN (16)]. As time progresses, more than the Two-stage target detection algorithm is needed to cope with the current high volume of detection requirements. To compensate for the problems in the detection speed of the Two-stage detection algorithm, Redmon et al. (17) introduced the You Only Look Once (YOLO) target detection algorithm, a separate CNN (18) model implementing an end-to-end one-step detection system after extensive research in 2016. Following the update from v1 to v5 (19–22), the YOLO targeting algorithm has gained popularity as the primary framework for target detection due to its more straightforward methods and faster speed than the two-stage target detection algorithm.

Although YOLO target detection algorithms can rapidly and effectively recognize images, they have some weak points. The most significant weakness is the inability to detect objects close to each other. This shortfall is related to the basic principles of the algorithm, and it has resulted in the algorithms not being commonly utilized in construction sites. The YOLO target detection algorithm divides an input image into S × S grids. If the center of an object falls into a grid cell, that cell is responsible for detecting the object. The YOLO target detection algorithm’s basic principles mean that predicting objects with overlapping parts can be challenging. Two ideas have been proposed to solve the problem of YOLO in target detection. The first idea is to use the SSD (23) target detection algorithm and multi-scale cells to improve the situation. For instance, Jiang et al. (24) have used a balanced feature pyramid structure and Global Correlation Network (GCNet) (25) to enhance the feature fusion and feature extraction capabilities of the YOLOv5 model. This approach can effectively deal with helmets with stains, partially obscured targets, and low-resolution images. The second idea is to adopt the Faster R-CNN target detection algorithm and anchor boxes. Chen et al. (26) proposed a face detection model based on YOLOv3, using anchor boxes that are more suitable for face detection and a more accurate regression loss function; this method can significantly increase precision while maintaining fast detection speed.

Based on the above considerations, a new idea for detecting small targets, such as PPE, has been proposed. In the past, YOLO target detection algorithms that were enhanced were typically based on CNN. CNNs are helpful for various computer vision tasks due to their inherent properties. However, they have some limitations, such as their ability to capture local information and their inability to establish long-distance connections. The Transformer, an attention-based encoder-decoder architecture, has been used in the popular chatbot model and has revolutionized the field of natural language processing (NLP) (27) in recent years. Additionally, it has made significant contributions to the field of Computer Vision. Compared with CNN, Transformer offers superior modeling capabilities and a powerful sensory field of view. Consequently, using Visual Transformer to improve the YOLOv5s model, we have improved YOLOv5s-g^n^Conv, YOLOv5s-HorBlock, and YOLOv5s-HorNet by combining the actual situation of construction sites and previous research. After a comparative analysis, the YOLOv5s-g^n^Conv performs surprisingly in terms of speed and accuracy of detection, allowing it to be applied accurately and efficiently to construction scenarios.

## 2. Materials and methods

### 2.1. Materials

#### 2.1.1. Image data acquisition

Firstly, we constructed the dataset by photographing on-site using field research methods. The location of the shooting was located in Guangdong Shengfeng Electric Power Engineering Co. in Guangzhou, Guangdong, China. The image data used were collected on March 12, 2023 at 10 a.m. and 3 p.m., under cloudy and sunny skies (workers at height rarely work on rainy days). We collected 1,000 images of working at height workers in four situations: repairing oil-immersed air-cooled transformers, climbing telephone poles, climbing pylons, and climbing steel platforms, 250 images for each situation. Considering that the camera’s view angle affects the detection performance, part of the images was acquired from multiple views during the image acquisition process. Among the 1,000 images, 296 were collected from different viewing angles, including 74 of workers repairing oil-immersed air-cooled transformers, 78 of workers climbing telephone poles, 72 of workers climbing pylons, and 74 of workers climbing steel platforms. These 1,000 images were expanded to 8,250 images using data enhancement methods.

Secondly, we use Python crawling techniques to get a large number of datasets on Google Images, the version of Python used was 3.8.10, and the crawler framework used was Scrapy (an open-source web crawler framework) written in the Python language, keywords such as “working at height,” “protection for working at height,” “safety protection for working at height,” “safety belts,” and “safety helmets” was searched, and 1,750 images of the standardized wearing of helmets and safety harnesses have been selected.

**Figure 1:**
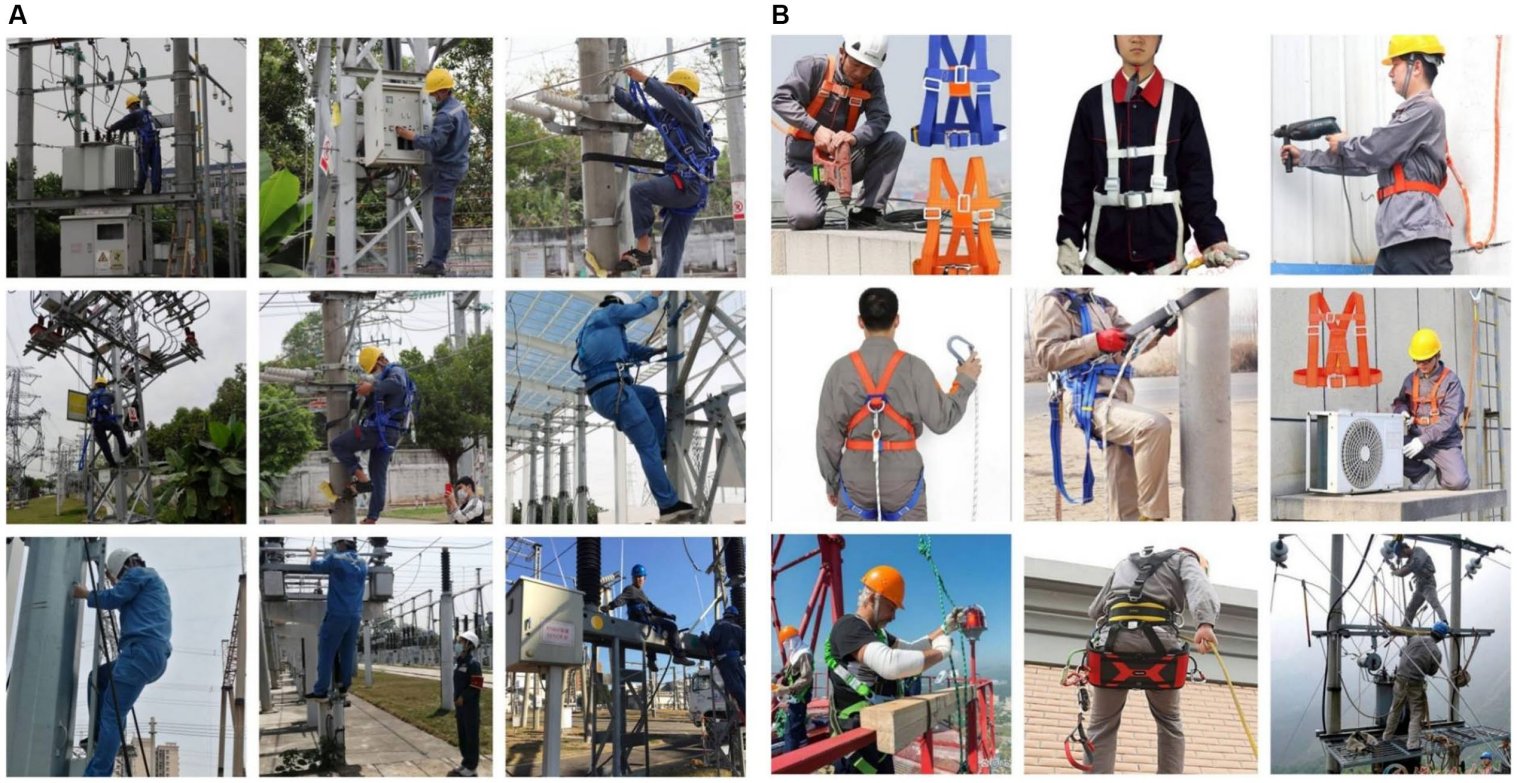
Image data acquisition: **(A)** Field photography and **(B)** Python crawler.

Finally, the two parts of data (a total of 10,000 sheets) were pooled to generate a dataset (Figure 1) which was used to train and test the detection model.

#### 2.1.2. Image data augmentation

**Figure 2:**
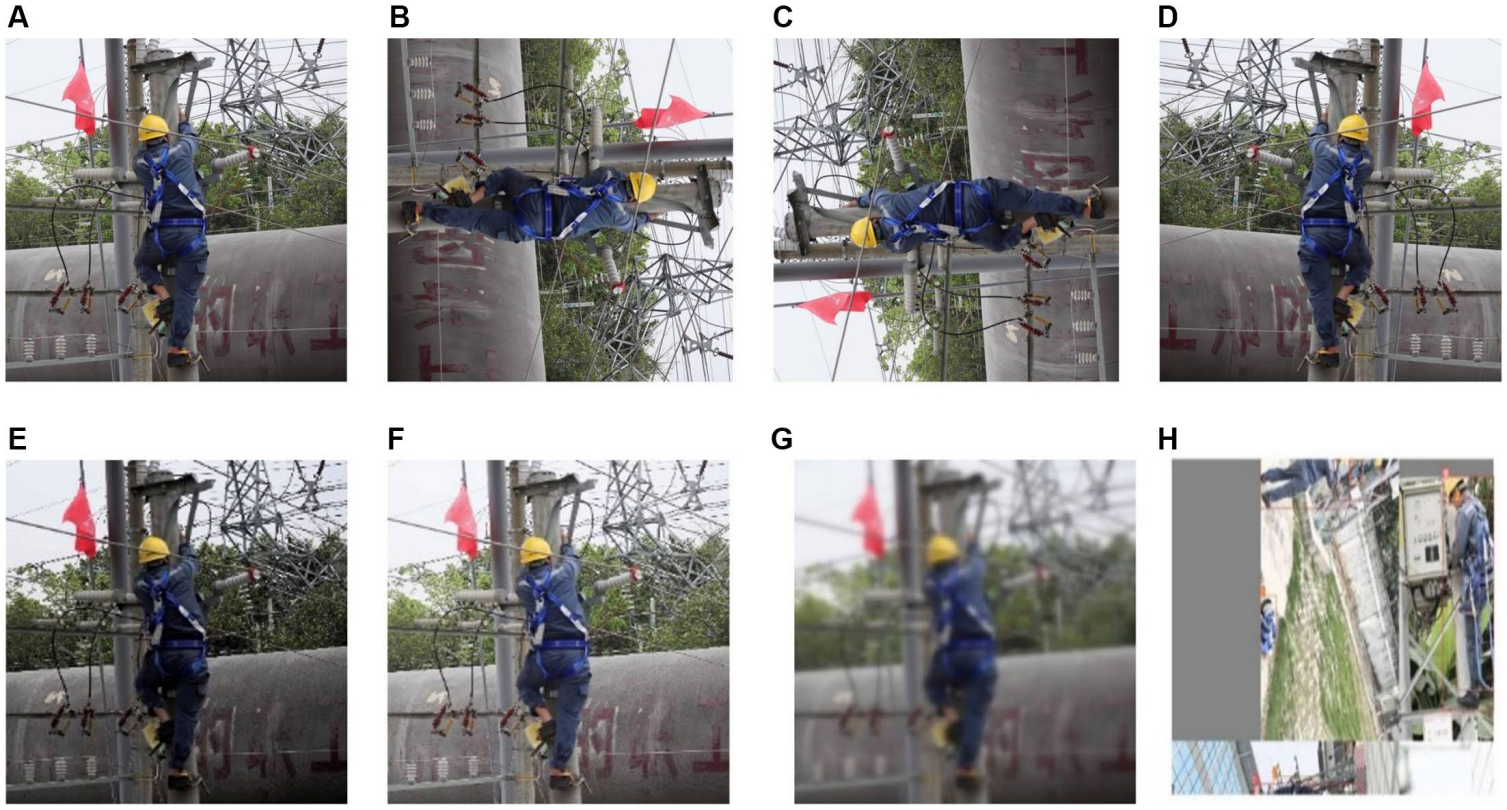
Image enhancement methods: **(A)** Original image, **(B)** 90° clockwise rotation, **(C)** 270° clockwise rotation, **(D)** Horizontal mirror, **(E,F)** Brightness transformation, **(G)** Blur processing, and **(H)** Image mosaic.

The neural network’s ability to process images taken at different times of the day depends on the quality of the training dataset since sunlight angle and intensity change throughout the day. To create a more comprehensive dataset that mimics the images seen by the human eye, we pre-processed the collected images by adjusting brightness, rotation, image sharpness, and mosaic effects. Figure 2 shows the augmented dataset.

##### 2.1.2.1. Image brightness

Image brightness change is based on Hue, Saturation, Brightness (HSB); HSB is based on the intuitive properties of color by A. R. Smith in 1978 to create a color space (28).To compensate for the drawback that neural networks are not robust to various light intensities due to the concentration of image acquisition time. The brightness of the images in the dataset is processed as follows: Two randomly selected values from L_min_ to L_max_ were used to adjust the brightness of the original image, and these three new results were added to the dataset. If the image brightness is too high or too low, causing the edges of the target to be unclear, the borders were difficult to draw when manually annotating. Thus, these imperfect training set images would have a detrimental effect on the performance of the detection model during training. In order to avoid producing such images, during manual annotation, we chose a suitable range of image luminance transformations, i.e., Lmin = 0.6 and Lmax = 1.4, based on whether we could accurately identify the target edges. This approach simulates the situation of a construction site under different light intensities.

##### 2.1.2.2. Image rotation

In Computer Vision, many pictures of objects are rotated 90, 180, 270, etc. in order for the machine to better simulate the real world as seen by the human eye (29).Original images were rotated by 90°, 270°, and mirrored, the detection performance of the neural network was also improved via the rotated image.

##### 2.1.2.3. Image blurring

The main purposes of image blurring is to give the image preprocessing to reduce the image noise. For example, to remove some trivial details from the image before the extraction of large targets (30).The image acquisition may be unclear due to the shooting distance, camera movement, and camera focus, which also affects the detection results of the neural network. Therefore, random blurring is applied to images enhanced by luminance and rotation to improve the robustness of the data.

##### 2.1.2.4. Image mosaic

Mosaic data enhancement method is proposed in YOLOv4 target detection algorithm (21), and the main idea is to randomly crop four images and splice them onto one image as training data. To reduce the risk of overfitting and improve the model’s performance and versatility, we augmented the original images using Mosaic data enhancement.

#### 2.1.3. Images annotation and dataset production

To better compare the performance of different algorithms, images in the dataset to YOLO format. When creating the dataset, the length of the dataset image is rescaled to 608 pixels, and the width is adjusted accordingly to maintain the original aspect ratio. The images are numbered and then manually labeled. Draw bounding boxes and manually classify categories. Samples with insufficient or unclear pixel areas are not labeled to prevent overfitting of the neural network. In the case of occlusion, targets with an occlusion area greater than 80% and targets with an image edge area less than 20% are not labeled. After labeling the data, we get the number of training sets: the number of validation sets: number of test sets =7,000:2,000:1,000. The training set serves the purpose of fitting the model’s parameters by utilizing the training samples. This set primarily facilitates the training of the neural network’s internal parameters. The validation set, kept separate during model training, is invaluable for hyperparameter tuning and an initial assessment of the model’s capabilities. Lastly, the test set plays a crucial role in evaluating the final model’s generalization ability. It provides insights into how well the trained model can perform on new and previously unseen data samples.

### 2.2. Methods

#### 2.2.1. YOLOv5s

**Figure 3:**
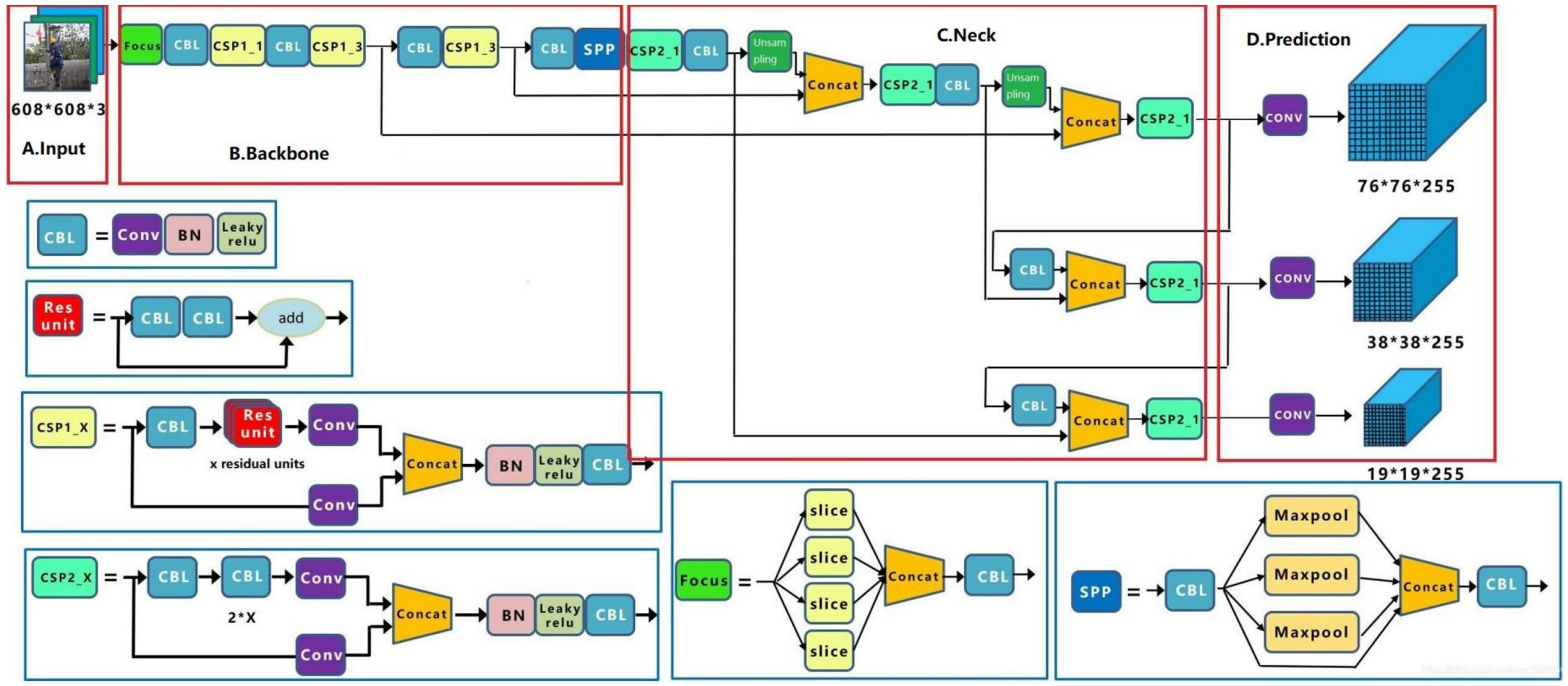
YOLOv5s structure: **(A)** Input, **(B)** Backbone, **(C)** Neck, and **(D)** Prediction.

The YOLOv5 is a group of models developed and maintained by Ultralytics (22). It includes YOLOv5s (minimum), YOLOv5m, YOLOv5l, and YOLOv5x (maximum), each with its benefits in terms of performance and suitability for different applications. Detecting PPE for workers at height requires real-time monitoring. The YOLOv5s model was chosen because it has the fewest neural network layers and the fastest inference speed, despite being slightly less accurate than other models. The YOLOv5s model comprises an input, backbone, neck, and output, and its overall block diagram can be seen using the network visualization tool, Netron (31) (Figure 3).

##### 2.2.1.1. Input

In step 1, datasets of varying sizes were scaled to uniform 608 × 608 × 3 images and sent into the detection network. Step 2, the adaptive anchor frame is computed. In this paper, the initial anchor frame of the COCO dataset (32) is chosen, the network output the predicted frame based on the initial anchor frame and compares it with the actual frame, calculated the difference between the two, and then updates it backward, iterating the network parameters. Step 3, Mosaic data enhancement, which randomly used four images, scaled randomly, and then randomly distributed them for stitching, enriching the detection dataset, especially random scaling added many small targets and improved the robustness of the network.

##### 2.2.1.2. Backbone

The backbone network of the YOLOv5s model consists of Focus, CSP1_X, and SPP networks. In step 1, the input image of 608 × 608 × 3 was sliced through the Focus module to reduce the height and width of the image and integrated by Concat to increase the number of channels of the input image, which is 64. In step 2, the integrated image was extracted by the Conv convolution module with size 3 and step size 2, and the output image size was 152 × 152 × 128. Step 3, after three sets of CSP1_3 and Conv convolution operation, a feature map with image size 19 × 19 × 1,024 was obtained. Step 4, the SSP module was used to perform 1 × 1, 5 × 5, 9 × 9, and 13 × 13 times maximum pooling operations on the 19 × 19 × 1,024 feature maps to extract features from various aspects and the four sets of pooled feature maps were aggregated by Concat to improve the model accuracy.

##### 2.2.1.3. Neck

YOLOv5s used CSP2_1 and CSP2_2 to increase network speed while maintaining accuracy. In this part, YOLOv5s used the CSP2 module to reduce the number of model parameters by upsampling 76 × 76 × 255 sized feature maps. In more detail, the upsampling process was the connection of two sets of CSP2, Conv with size 1 and step size 1, Upsample and Concat. More specifically, the *j* in Concat [*i*, *j*] represents the feature map obtained from the *j*th layer operation in the network. Then, The 76 × 76 × 255 feature map was downsampled to obtain 76 × 76 × 255, 38 × 38 × 255, and 19 × 19 × 255 feature maps, the three different sizes.

##### 2.2.1.4. Prediction

Generate candidate boxes on three different scale feature maps; next, GIOU_Loss (33) and non-maximum suppression (NMS) (34) was applied, and finally, the target boxes were filtered to generate output target classification and border regression.

#### 2.2.2. YOLOv5s-HorBlock and YOLOv5s-HorNet

YOLOv5s-HorBlock introduces a horizontally connected network structure based on the YOLOv5s model, which allows for more semantic information and contextual connections by horizontally connecting feature maps at different levels. This horizontally connected approach helps to improve spatial interaction modeling and can better capture relational information between different targets. Since there are various interactions between workers and the environment in a construction site, such as buildings, equipment, and people, the correlations between them can be better understood through horizontal connectivity. For example, horizontal connectivity can be utilized better to handle the interaction between safety equipment and personnel, and to determine whether or not there is a norm to wear personal protective equipment.

The YOLOv5s-HorNet structure uses an hourglass network that enables target detection through multi-scale feature maps, further enhancing the model’s perception and modeling capabilities. This helps to solve the problem of scale variations present in construction sites, such as detecting both near and far targets. In addition, HorNet can better handle occlusion situations in building structures and improve the model’s ability to understand complex scenes.

YOLOv5s-HorBlock, YOLOv5s-HorNet, and YOLOv5s-g^n^Conv have the advantages of more robust spatial interaction capability, higher accuracy, and faster thrust speed compared to YOLOv5s-g^n^Conv, which is also verified in the subsequent experiments.

#### 2.2.3. YOLOv5s-g^n^Conv

According to the progress of research on Vision Transformers (35), driven by a new spatial modeling mechanism based on dot-product self-attention, which can achieve significant breakthroughs in various tasks, and the critical components behind visual converters, i.e., input adaptive, long-range and higher-order spatial interactions, which can be efficiently implemented by a convolution-based framework, are invoked to combine recursive gated convolution (g^n^Conv) (36) with the YOLOv5s algorithm, which combines the advantages of Vision Transformers and CNN, naming it YOLOv5s-g^n^Conv,YOLOv5s-g^n^Conv achieves efficient higher-order spatial interaction modeling based entirely on the convolutional structure through g^n^Conv and loop design, which is compatible with various convolutional forms and can extend the second-order spatial interaction modeling to arbitrary order without significantly increasing the computational effort.

##### 2.2.3.1. Spatial modeling operations

**Figure 4:**
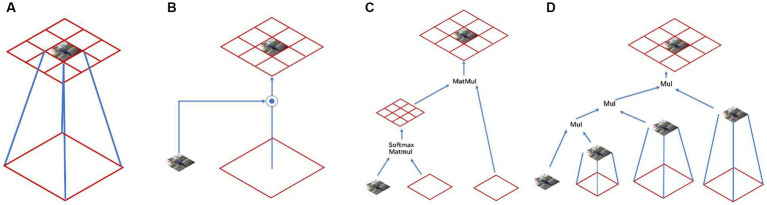
Different kinds of spatial modeling: **(A)** Standard convolution, **(B)** Dynamic convolution, **(C)** Self-attention convolution, and **(D)** gnConv.

Different model structures have different abilities to model spatial interactions, and the expressiveness of the model can be improved by increasing the order of spatial interactions (36). Standard convolution did not take into account spatial interaction information (Figure 4A); Squeeze-and-excitation networks (37) and dynamic convolution (38) took into account the information interaction in the surrounding region with the help of dynamic weights, which made the model more capable (Figure 4B); the Self-attention part achieved second-order spatial information interaction by multiplying two consecutive matrices between query, key, and value (Figure 4C). Specifically, the method proposed in this paper can efficiently achieve information interaction of arbitrary order with the help of g^n^Conv and recursive operations (Figure 4D).

The receptive field is one noticeable difference between the Vision Transformer and traditional CNNs. While traditional CNNs typically use 3 × 3 convolution over the entire network, the Visual Transformer uses 7 × 7 convolution over the entire feature map to compute self-attention (39). The receptive field in Vision Transformer makes it easier to capture long-term dependencies (40), which is one of the core advantages of Vision Transformer.

##### 2.2.3.2. YOLOv5s-g^n^Conv structure

**Figure 5:**
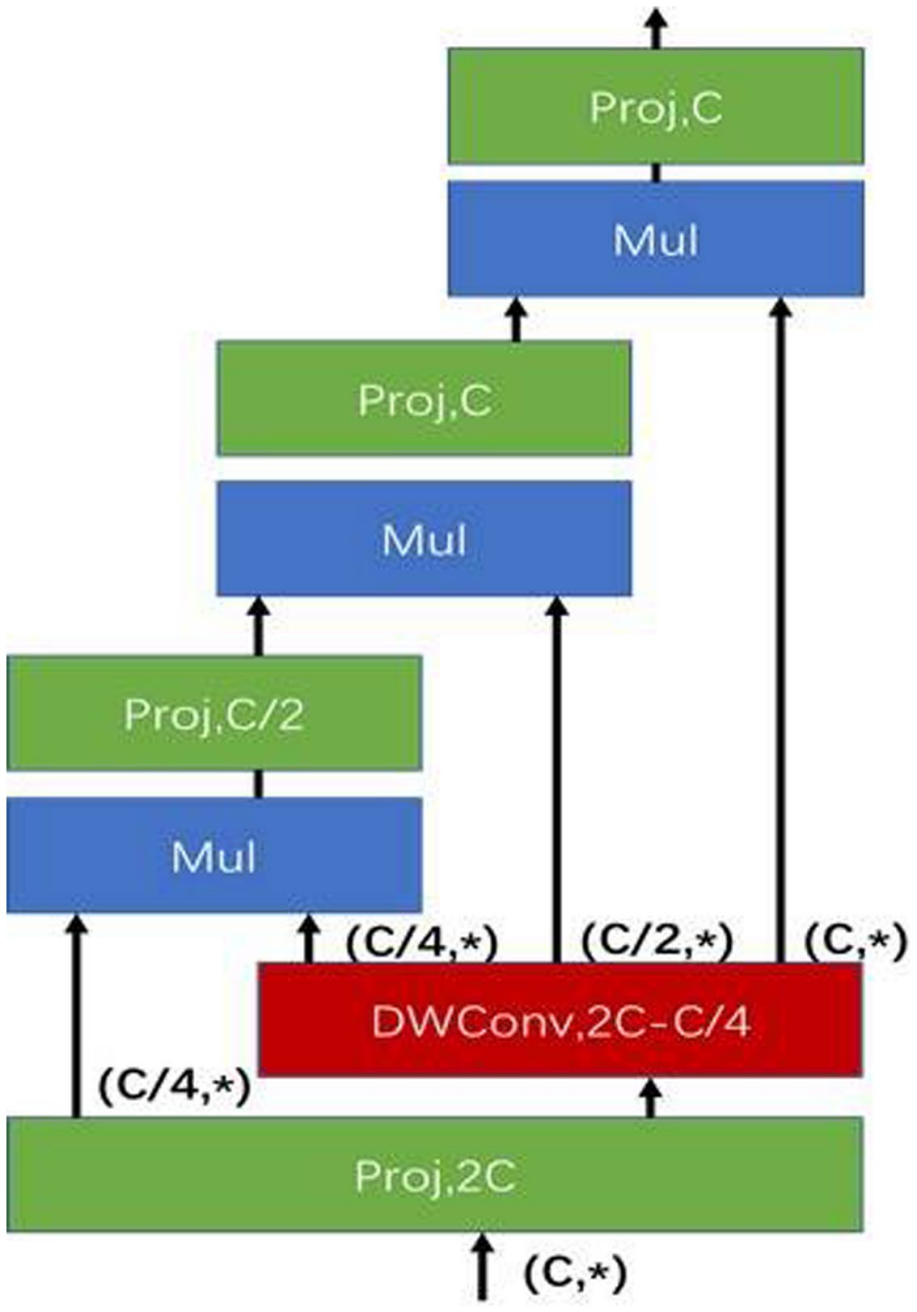
Model structure of g^3^Conv (Third-order g^n^Conv model).

YOLOv5s target detection algorithm uses the structure of FPN (41) and PAN (42) for the multi-scale fusion of features. g^n^Conv (Figure 5) instead of the spatial convolution used for feature fusion in FPN. Due to the extensive use of convolutional models in the YOLOv5s target detection algorithm, g^n^Conv is added after fusing features from different pyramid levels, and the introduced g^n^Conv takes into account higher-order spatial interactions to improve the spatial interaction between upstream and downstream tasks.

**Figure 6:**
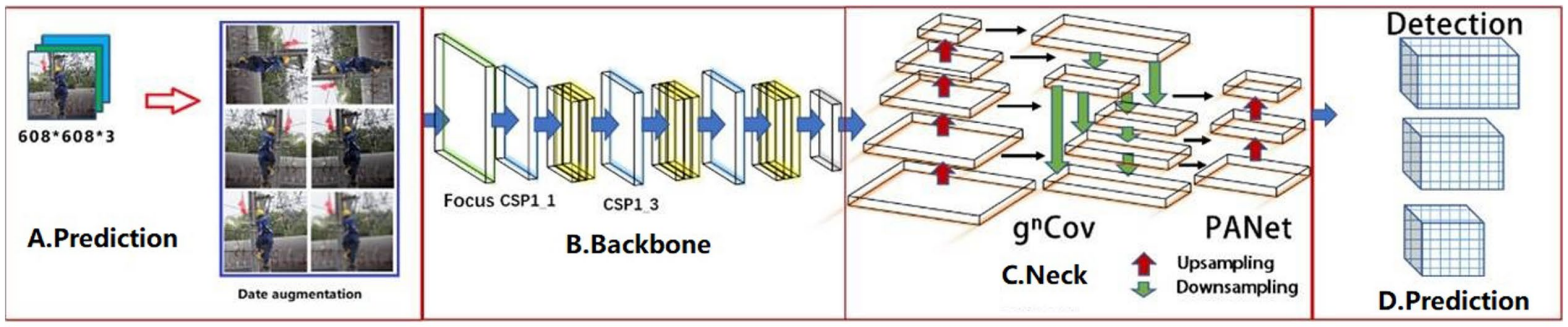
YOLOv5s-g^n^Conv structure: **(A)** Input, **(B)** Backbone, **(C)** Neck, and **(D)** Prediction.

Next, compare the differences between YOLOv5s-g^n^Conv (Figure 6) and YOLOv5s. The backbone and Prediction parts of the YOLOv5s-g^n^Conv network structure are the same as those of the YOLOv5s network structure and will not be described in detail here.

###### 2.2.3.2.1. Input

The YOLOv5s-g^n^Conv target detection algorithm significantly changes in its input side. It eliminates the mosaic enhancement method used in the original network and opts for the data enhancement method in Section 2. This new method pre-processes the dataset for brightness, rotation, blurring, and Mosaic. The data enhancement techniques not only expand the dataset but also improve the quality of data samples, resulting in an enhanced model generalization ability.

###### 2.2.3.2.2. Neck

To enhance detection performance for multi-scale targets while ensuring real-time detection, g^n^Conv is used instead of spatial convolution for feature fusion in FPN. Additionally, each layer’s g^n^Conv is used instead of the 3 × 3 convolution on the top-down path to improve spatial interaction for downstream tasks.

##### 2.2.3.3. Computational complexity of gated convolution of high order

The calculation of g^n^Conv can be divided into three parts. Projection layer $\emptyset_{\mathrm{in}}$ in the first step and projection layer $\emptyset_{\mathrm{out}}$ in the last step:

$$\mathrm{FLOPs}\left(\emptyset_{\mathrm{in}}\right)=2HWC^{2}$$

$$\mathrm{FLOPs}\left(\emptyset_{\mathrm{out}}\right)=HWC^{2}$$

where, $\emptyset_{\mathrm{in}}$ , $\emptyset_{\mathrm{out}}$ is the linear projection operation to complete the information exchange in the channel dimension. H is the height of the image, W is the width of the image.

Depth-wise convolution f: Kernel sizes means that the Depth-wise convolution of K × K acted on the feature ${\left\{q_{k}\right\}}_{k=1}^{n-1}$ , $q_{k}\in R^{HW\times C_{k}}$ , $C_{k}=\frac{C}{2^{n-k-1}}$ . Therefore, the computation of this part is as follows:

$$\mathrm{FLOPs}\left(\mathrm{DWConv}\right)=HWK^{2}\overset{n-1}{\underset{k=0}{\sum }}\frac{C}{2^{n-k-1}}=2HWCK^{2}\left(1-\frac{1}{2^{n}}\right)$$

Computation of g in dimensional matching in recursive gating operations:

$$\begin{array}{l} \mathrm{FLOPs}\left(\mathrm{RecursiveGating}\right)=HWC_{0}\\ +\overset{n-1}{\underset{k=1}{\sum }}\left(HWC_{k-1}C_{k}+HWC_{k}\right)=HWC\left[\frac{2}{3}C\left(1-\frac{1}{4^{n-1}}+2-\frac{1}{2^{n-1}}\right)\right] \end{array}$$

Consequently, the total amount of calculations is as follows:

$$\begin{array}{l} \mathrm{FLOPs}\left(g^{n}\mathrm{Conv}\right)=\\ HWC\left[2K^{2}\left(1-\frac{1}{2^{n}}\right)+\left(\frac{11}{3}-\frac{2}{3\times 4^{n-1}}\right)C+2-\frac{1}{2^{n-1}}\right] \end{array}$$

$$\mathrm{FLOPs}\left(g^{n}\mathrm{Conv}\right)<HWC\left(2K^{2}+\frac{11}{3}\times C+2\right)$$

## 3. Results

### 3.1. Prerequisite

#### 3.1.1. Assumptions

The hypothetical detection scenario of this experiment: under standard construction, the density of overhead workers is moderate. If the construction scene is dimly lit, the target detection algorithm cannot accurately recognize the target, resulting in detection failure; if the density of overhead workers is too high, resulting in the detection of target overlap is too high, which will also lead to detection failure.

#### 3.1.2. Environment

The model training platform uses The 12th Gen Intel® Core™ i5-12500H processor; The GPU is an NVIDIA GeForce RTX 3060 graphics card with 16 GB (4,800 MHz) of video memory; the operating system is Windows 11 ProPlus; and the model training is performed on the PyTorch deep learning framework.

#### 3.1.3. Metrics

Commonly used target detection metrics were Intersection over Union (IoU), Precision, Recall, mean Average Precision (mAP), and Frames Per Second (FPS).

Intersection over Union was a metric of the degree of overlap between two regions, as follows:

$$IoU=\frac{\mathrm{Area}\;\mathrm{of}\;\mathrm{Overlap}}{\mathrm{Area}\;\mathrm{of}\;\mathrm{Union}}$$

IoU ≥ 0.5, which meant that the model detection overlaps the actual object border by more than 50%, was considered True Positive (TP). IoU < 0.5, which meant that there was too little overlap between the border of the detected object and the actual border, was considered False Positive (FP). If the object was there and the model did not detect it, it was considered False Negative (FN). If the object did not exist and the model did not detect it, it was considered True Negative (TN).

The formulae for the precision, recall, and accuracy indicators could be obtained based on the confusion matrix.

Precision was the proportion of positive boxes inferred by the True Positive model. In other words, it was an indicator for assessing the accuracy of a model’s predictions.

$$\mathrm{Precision}=\frac{TP}{TP+FP}$$

Recall indicates how many real target objects the model has successfully reasoned about. In other words, it was an indicator for assessing the completeness of a model’s inference on natural target objects.

$$\mathrm{Recall}=\frac{TP}{TP+FN}$$

mAP indicated how good the learned model was across *N* categories, then averaged over *N* categories in terms of average precision (AP).

$$\mathrm{mAP}=\frac{AP}{N}$$

Frames Per Second was the number of images that could be processed per second.

$$FPS=\frac{\mathrm{frameNum}}{\;\mathrm{elapsedTime}}$$

#### 3.1.4. The loss function

The basic idea of YOLOv5s target detection algorithm is to divide the 608*608 input image into N*N grids and then predict three metrics for each grid of the grid: rectangular box, confidence level, and classification probability. The rectangular box characterizes the size and precise location of the target; the confidence level characterizes the degree of confidence of the predicted rectangular box (referred to as the prediction box), with values ranging from 0 to 1; the larger the more significant indicates that the target is more likely to exist in the rectangular box; and the classification probability characterizes the category of the target.

The loss function measures of the distance between the neural network’s predicted information and the desired information (labeling); the closer the predicted information is to the desired information, the smaller the loss function value is. The training contains three main aspects of loss: rectangular box loss (lossrect), confidence loss (lossobj), and classification loss (lossclc). Therefore the loss function of the YOLOv5s network is defined as:

Loss=a∗lossobj+b∗lossrect+c∗lossclc

The Loss is the weighted sum of the three losses, with the confidence loss usually taking the most significant weight and the rectangular box loss and the classification loss having the next highest weights.

### 3.2. Comparison of different algorithms

To test the superiority of the model described in this paper, images of workers wearing personal protective equipment while working at heights were used as the dataset. The YOLOv5s-g^n^Conv model proposed in this paper was found to be superior to other popular target detection models such as YOLOv5s and Faster R-CNN network models, as well as two other improved algorithms, YOLOv5s-HorBlock and YOLOv5s-HorNet, based on both horizontal and longitudinal comparisons.

To easily compare various algorithms, the training parameters for each network model were uniformly set. The training set sample batch size was 32, and eight workers were assigned. Three hundred iteration cycles were completed, with all other coefficients set to default values. We used a visualization tool for deep learning-Wandb to obtain the experimental data.

**Figure 7:**
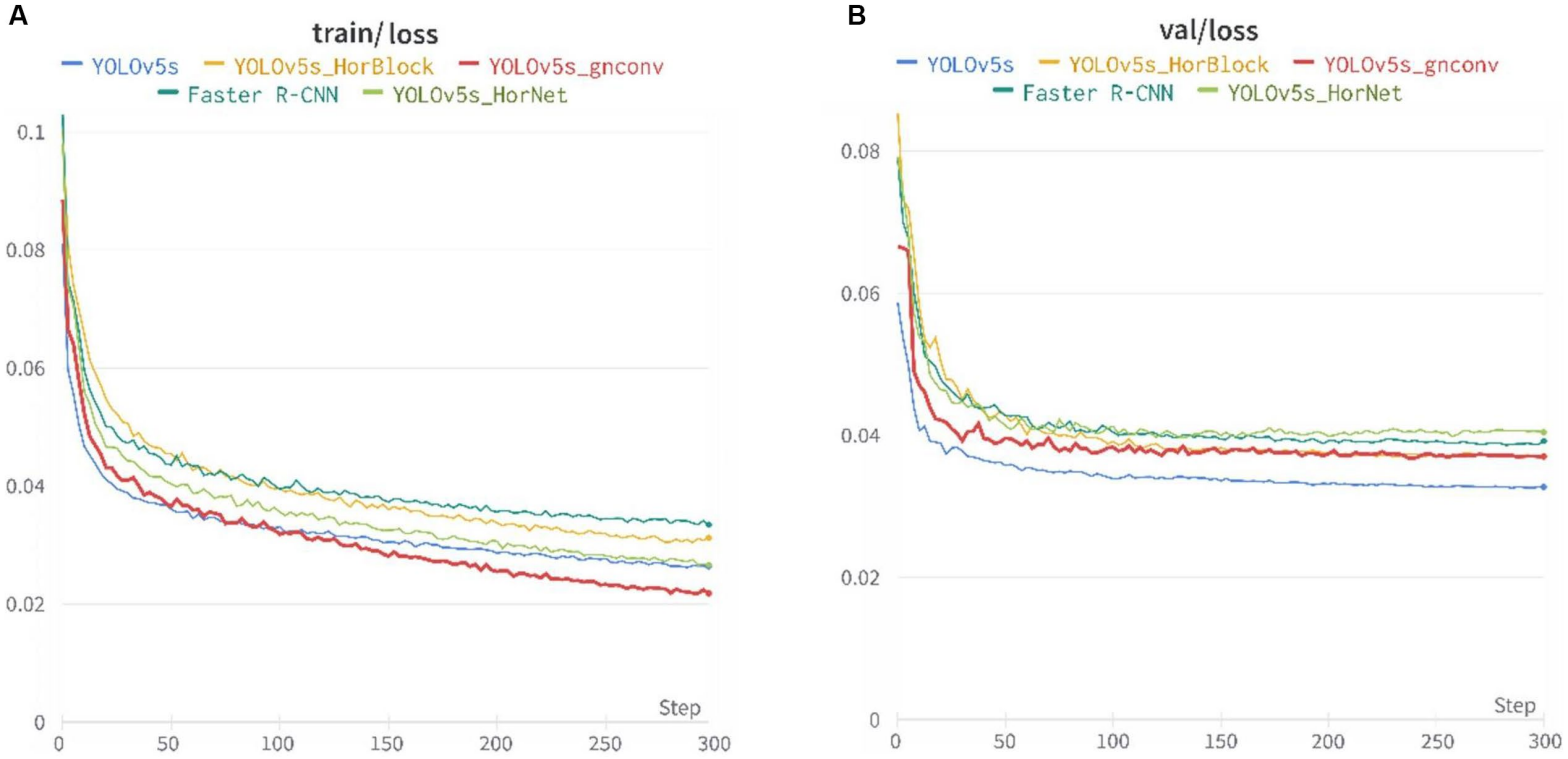
Loss curves of the five models: **(A)** Training set loss function and **(B)** Validation set loss function.

The training loss function and the validation loss function after 300 iteration cycles of training are shown in Figure 7. The loss gradually decreased and converged with the increase of steps, the loss function decreased fastest in the first 50 cycles, and the training loss function continued to decrease slowly in the later training, where YOLOv5s-g^n^Conv decreased fastest, representing the superiority of the model in the learning phase; the loss of the validation loss function tended to stabilize and converge to a specific value in the later training.

**Figure 8:**
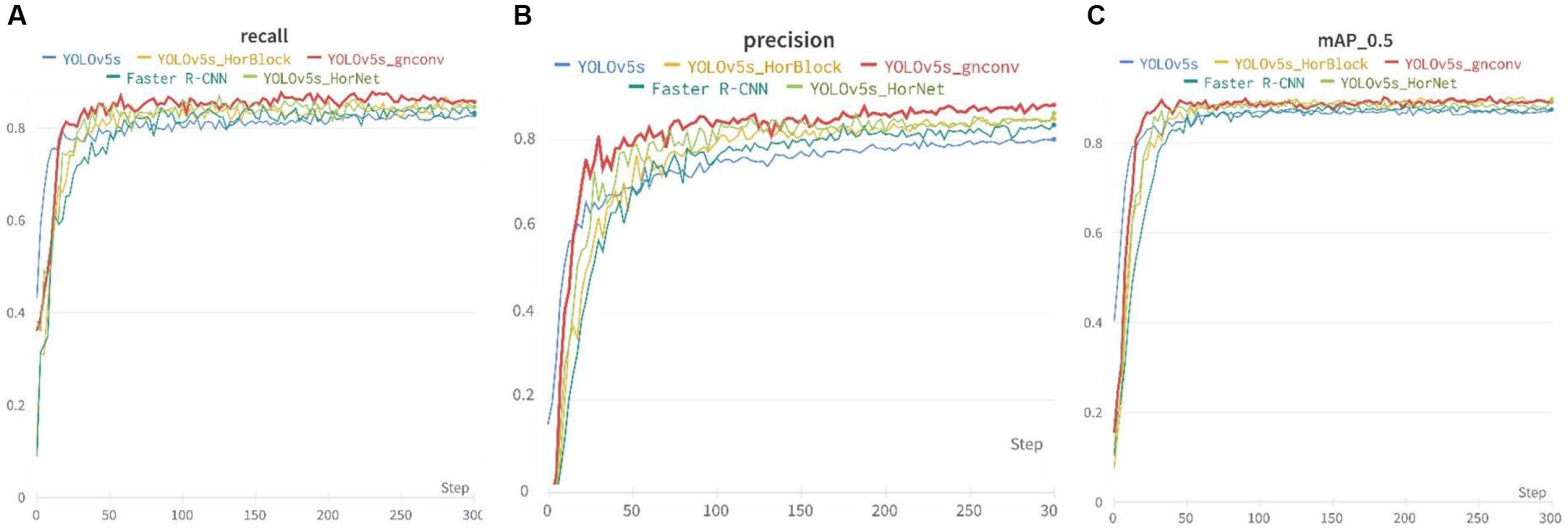
Recall, mAp_0.5, and precision of the five models: **(A)** Recall, **(B)** Precision, and **(C)** mAP_0.5.

Figure 8 presents the recall, mAP_0.5, and precision metrics for the training dataset were presented. Specifically, the recall and mAP_0.5 remained stable after around 50 cycles during the initial stages of training, while the precision gradually rose and stabilized in the middle and later stages of training.

Table 1 above displays the detection results. YOLOv5s-g^n^Conv showed noteworthy improvements when compared to the official YOLOv5s model. In recall, mAP_0.5, and precision metrics, YOLOv5s-g^n^Conv had a 4.72% improvement in recall, a 4.26% improvement in mAP_0.5, and a 5.01% improvement in precision. Additionally, the detection speed of YOLOv5s-g^n^Conv was 17.75% faster than YOLOv5s. These outcomes demonstrate the proposed model’s superior performance.

**Table 1:** Experimental data of the five models.

Models	Recall	mAP_0.5	Precision	FPS
YOLOv5s-g^n^conv	89.37%	92.96%	97.32%	56.12f/s
YOLOv5s-HorBlock	87.23%	91.45%	95.28%	49.32f/s
YOLOv5s-HorNet	86.95%	91.18%	95.36%	48.75f/s
Faster R-CNN	86.24%	90.45%	93.44%	52.14f/s
YOLOv5s	84.65%	88.70%	92.31%	47.66f/s

**Figure 9:**
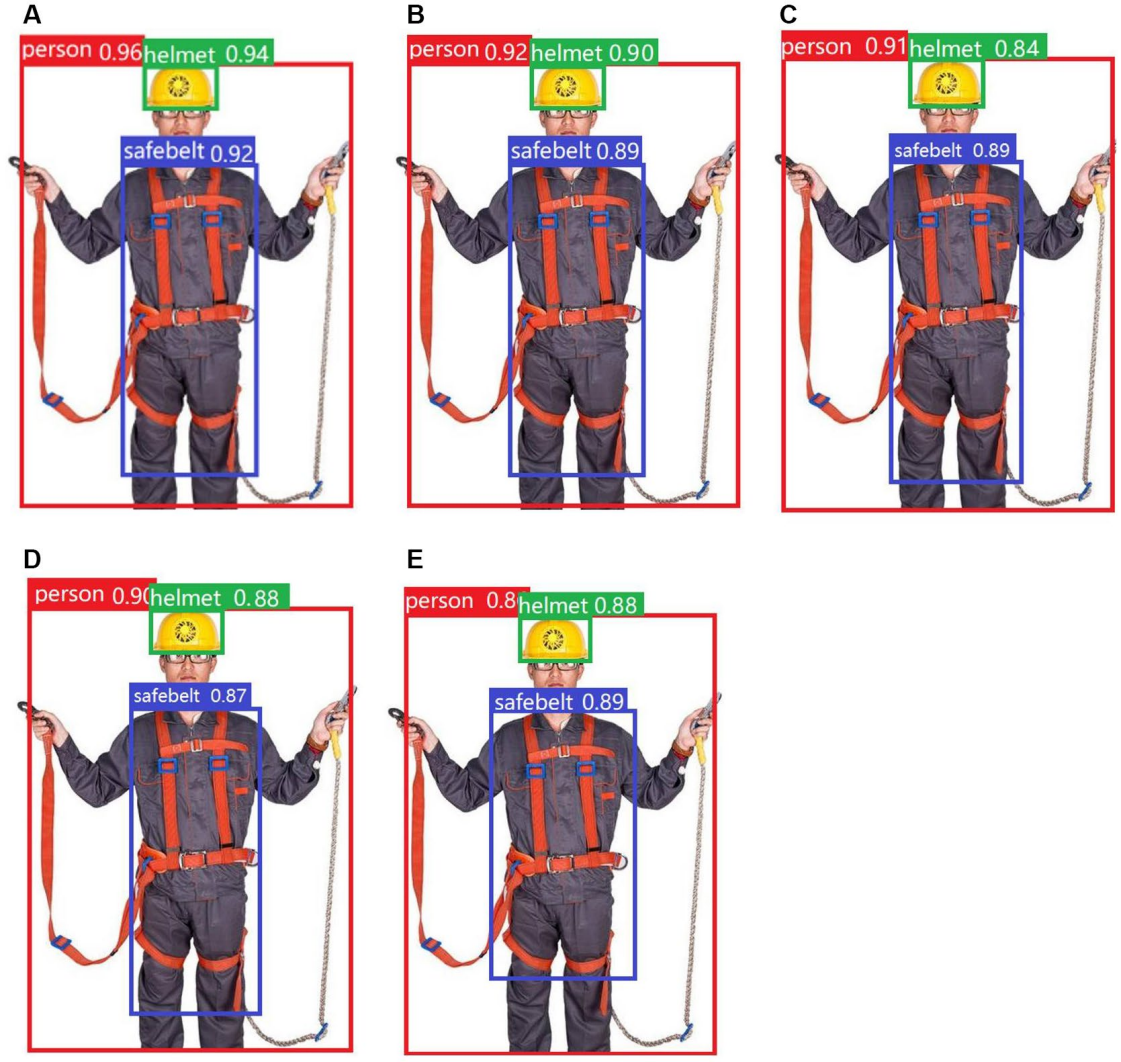
Detection results of the five models: **(A)** YOLOv5s-gnconv, **(B)** YOLOv5s-HorBlock, **(C)** YOLOv5s-HorNet, **(D)** Faster R-CNN, and **(E)** YOLOv5s.

Various models, including YOLOv5s-g^n^Conv, YOLOv5s-HorBlock, YOLOv5s-HorNet, Faster R-CNN, and YOLOv5s, were utilized to detect test images. The results of the detection process are displayed in Figure 9.

During the detection process, the YOLOv5s-g^n^Conv model provided significantly higher accuracy and confidence than the other four models, reflecting the superiority of the YOLOv5s-g^n^Conv detection model.

### 3.3. Ablation experiments

In order to further validate the detection performance of the algorithms proposed in this study and to explore the effectiveness of each improvement method, eight groups of ablation experiments were designed based on YOLOv5s. Each group of experiments used the same hyper-parameters as well as the training technique, with an initial learning rate of 0.01, a momentum parameter of 0.92, BatchSize of 64, 120 iterations of training, and the evaluation metrics: parameters, GFlops (Floating Point Operations, Flops is used to measure the time complexity of an algorithm), precision, and mAP_0.5. We used a visualization tool for deep learning-Wandb to obtain the experimental data. The experimental results are shown in Table 2.

**Table 2:** Ablation experiments.

Groups	g^n^Conv	HorBlock	HorNet	Parameters	GFlops	precision	mAP_0.5
1	×	×	×	7,114,785	16.5	88.7	82.40%
2	✓	×	×	5,976,401	16.8	90.46	86.35
3	×	✓	×	6,125,863	16.9	88.98	83.89
4	×	×	✓	5,923,145	16.8	87.25	83.11
5	✓	✓	×	12,659,184	22.8	81.34	79.35
6	✓	×	✓	13,095,150	20.2	82.96	80.33
7	×	✓	✓	16,413,156	19.2	51.2	44.62
8	✓	✓	✓	16,780,348	24.6	53.28	48.23

Where g^n^Conv, HorBlock, and HorNet are mentioned in this study, “√” means that the module is introduced, and “×” means that the group of modules is not introduced.

As can be seen in Table 2, the introduction of the g^n^Conv, HorBlock, and HorNet modules reduces the amount of network computation by about 22% and the number of parameters by about 16%, which is an effective means of lightweight; After adding the g^n^Conv module alone, precision improves by 1.76%, and mAP_0.5 improves by 3.96%, which improves the accuracy and performance of the model. This proves that the introduction of the g^n^Conv module utilizes higher-order spatial interactions to improve the accuracy of multi-scale target detection and recognition, which is very effective for personal protective equipment with many overlapping parts of the target detection objects and requires more accurate localization. Protection products, the inclusion of g^n^Conv is very effective; Adding the HorBlock module alone improves precision by 0.28% and mAP_0.5 by 1.49%, which is not a significant improvement in network accuracy and performance, but it demonstrates that the horizontally-connected network structure introduced in the HorBlock module is working; Adding the HorNet module alone improves precision by 1.45% and mAP_0.5 by 1.71%, demonstrating that the Hourglass network used in HorNet is having an effect, which helps to solve the problem of scale variation that exists in construction sites, such as detecting both near and far targets. However there is little increase in the model’s accuracy and performance. After adding any two modules of g^n^Conv, HorBlock, and HorNet at the same time, the precision and mAP_0.5 are decreased to different degrees. The parameters of the model and the amount of computation are increased, especially when adding g^n^Conv and HorNet; at the same time, the precision and performance of the model are decreased the most, mainly due to the focus of the two modules’ perception. The main reason is that the two modules perceive different regions, g^n^Conv focuses on the global content, while HorNet focuses on local details. The merging of the two modules leads to more parameters accessible to overfitting, so the accuracy and performance have decreased significantly.

All three of g^n^Conv, HorBlock, and HorNet are lightweight modules that reduce the network complexity, while g^n^Conv balances between both speed and accuracy. The final improved YOLOv5s-g^n^Cnov model has a more significant improvement in accuracy and performance than YOLOv5s.The model’s ability to fit the target frame is further strengthened, which can be widely used in detecting a PPE for workers working at hight.

## 4. Discussion

**Figure 10:**
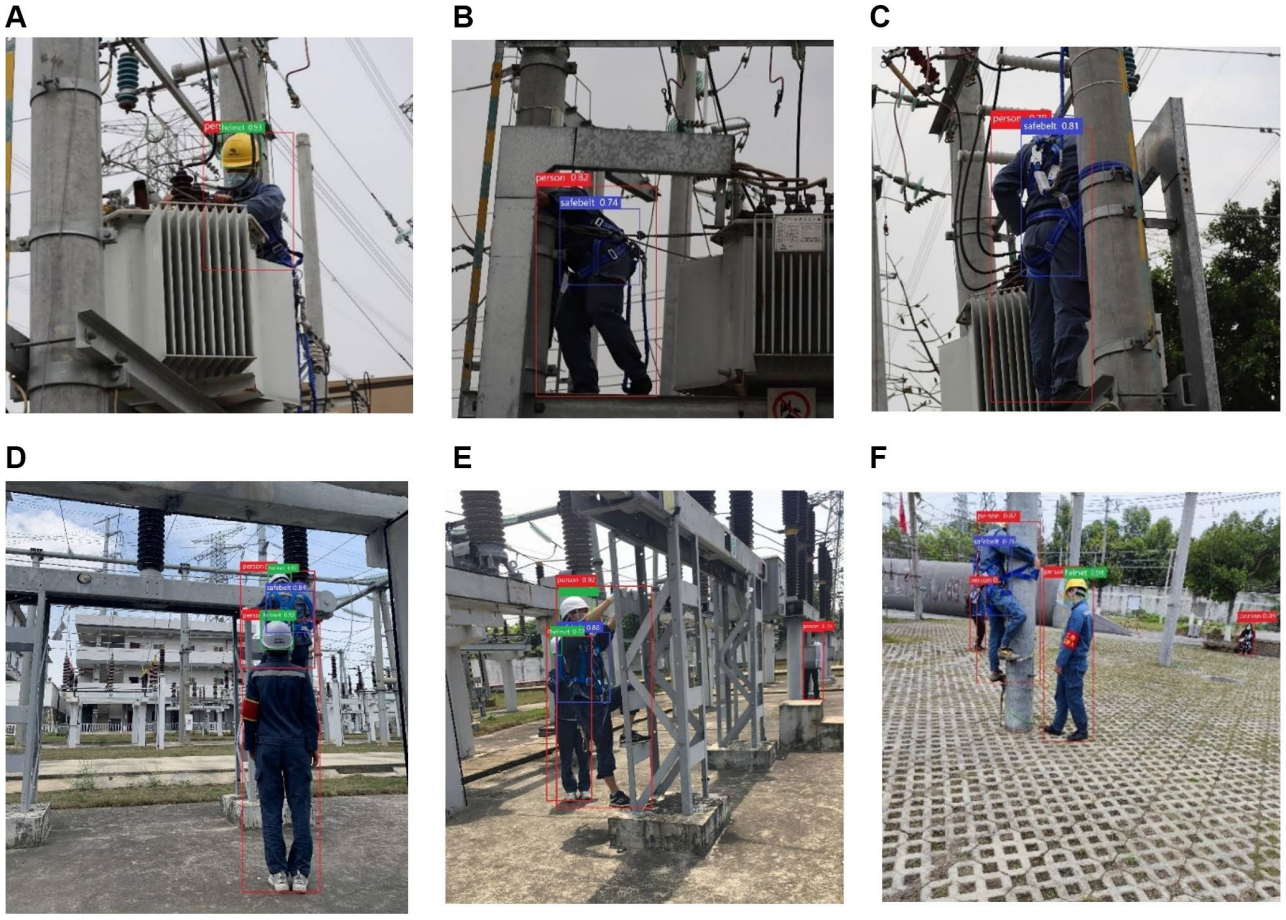
Detection results for occluded and overlapped PPEs: **(A)** Occluded part of the body, **(B)** Occluded part for head(side), **(C)** Occluded area is head (back), **(D)** Overlapped part of the body, **(E)** Overlapped part of the head(side), and **(F)** Overlapped part of the head(back).

Deploying the YOLOv5s-g^n^Conv model in natural construction site environments can effectively monitor and manage the safety of workers at height. However in practice, we need to consider the impact and potential limitations of the following aspects: lighting conditions, camera angles, and worker movement patterns (Figure 10).

### 4.1. Lighting conditions

Lighting conditions are one of the most critical factors affecting testing effectiveness. In construction site environments, especially in high-rise work areas, three conditions may occur: excessive lighting, insufficient lighting (in the case of nighttime construction), and uneven lighting. This will impose some limitations on the visual analysis of the YOLOv5s-g^n^Conv model and may lead to degradation of image quality and difficulty in target detection. To overcome these three problems, we provide some practical measures.

When there is too much light, the image captured by the camera may be overexposed, resulting in the loss of target details and thus affecting the detection accuracy of the model. For this situation, consider installing light protection devices, such as sunshades or filters, in the high-level operation area to attenuate light intensity so that the camera captures adapted images.

Under insufficient light conditions, the images may become dark, and the details are unclear, affecting the model’s ability to detect and recognize the target. To cope with this problem, consideration can be given to adding additional lighting equipment, such as flares or spotlights, to be applied to high-level operational areas to provide sufficient illumination to capture clear images.

In addition, a reasonable lighting layout is also essential, which should be based on the characteristics and needs of the operation area to maximize image quality and target detection; in a construction site environment, the light distribution may be uneven due to the presence of building structures and obstacles, resulting in some areas being too bright and some areas being too dim. This uneven lighting condition can also negatively affect the detection effect of the model. To overcome this problem, the installation position and angle of the camera can be rationally selected according to the site’s layout and actual situation to capture the working area’s image as evenly as possible.

In order to overcome the impact of lighting conditions on the YOLOv5s-g^n^Conv model detection effect, we can take measures such as increasing lighting equipment and optimizing the lighting layout. This will ensure that the camera captures high-quality, clear images in the construction site environment, especially in the high-rise work area, thus improving the accuracy and reliability of model inspection.

### 4.2. Camera angles

The choice of camera angle is critical to monitoring workers at height. The correct camera angle provides a complete view and reduces dead spots. However, in high-rise construction sites, camera mounting locations may be limited due to constraints and construction structures, making achieving a perfect field of view difficult. With this challenge in mind, we offer three effective measures.

#### 4.2.1. Multi-camera layout

Use multiple cameras to form a camera network to cover a wider area. Cameras can be selected to be installed at different angles and orientations to provide a full range of views. By complementing the field of view of multiple cameras with each other, surveillance blind spots can be reduced, and the trajectory and behavior of workers at height can be better captured.

#### 4.2.2. Optimization of camera height and angle

When installing a camera, the height and angle of the camera should be optimized for the best view. Cameras should be installed at a suitable height that can cover the activity area of the worker at height and can minimize dead spots. By carefully planning and adjusting the angle of the cameras, we can ensure that the surveillance system provides a full and accurate view.

#### 4.2.3. Field of view analysis and assistive technologies

Field of view analysis and assistive technologies can help optimize surveillance results. For example, using video analytics algorithms, a camera’s field of view can be analyzed and evaluated in real-time to identify possible blind spots or monitoring gaps, and timely measures can be taken to correct them. Surveillance assistance using augmented reality (AR) technology or drones can provide a more comprehensive and accurate field of view.

In order to compensate for the lack of field of view brought about by the limitation of camera angles, measures such as multi-camera layouts, optimization of camera heights and angles, and the use of the field of view analysis and assistive technologies can be adopted. These measures will help improve the accuracy and completeness of surveillance of workers at height and ensure that their safety is effectively monitored.

### 4.3. Worker movement patterns

The movement patterns of workers in a construction site can also impact the surveillance system’s effectiveness. Workers will move freely through a construction site without being restricted to a specific path or area. This can lead to difficulties for the YOLOv5s-g^n^Conv model in tracking and recognizing workers in real-time, and to address this problem, we provide two approaches.

#### 4.3.1. Sensor technology

Sensor technology can be used to monitor the location and activities of workers, such as utilizing RFID tags, Bluetooth, or infrared sensors to locate and track workers in real-time. By arranging sensor devices inside the construction site, the location information of workers can be collected and connected to the monitoring system to update the location and status of workers in real-time. When workers go beyond the predefined activity area, the system can instantly send out alarms and take corresponding safety measures.

#### 4.3.2. Real-time monitoring and alarm system

Equipped with a real-time monitoring and alarm system, it ensures a quick response to abnormal worker activity or an emergency. By integrating video surveillance, voice recognition, image analysis, and other technologies, the system can analyze workers’ behaviors and movements in real-time and identify unsafe behaviors or dangerous situations. Once an abnormality is detected, an alert can be immediately sent to the relevant personnel, triggering emergency response measures such as notifying site management, automatically shutting down equipment, or initiating safety emergency procedures.

The impact of free movement patterns of workers on the effectiveness of surveillance systems can be effectively addressed by utilizing sensor technology and implementing real-time monitoring in construction sites. These measures can monitor and limit workers’ movement areas, provide more accurate surveillance results, and ensure that the safety of workers at height is effectively ensured.

## 5. Conclusion

In this study, we utilized the YOLOv5s-g^n^Conv model for personal protective equipment (PPE) detection for workers working at heights and achieved the following key findings and contributions: we successfully trained and applied the YOLOv5s-g^n^Conv model, which demonstrated excellent detection performance in the PPE detection task. The YOLOv5s-g^n^Conv model provides higher accuracy and faster inference than conventional models. The new aspect of this model lies in introducing of the g^n^Conv layer, which further improves the feature extraction capability and perception. Our experimental results show that the YOLOv5s-g^n^Conv model can accurately detect dangerous behaviors and safety hazards in construction sites, providing site managers with a real-time and effective means of safety monitoring. This is essential for preventing accidents and protecting workers’ lives.

However, there are still some potential challenges and future research directions when applying the model to natural construction site environments. First, the deployment of the model needs to take into account the hardware resources and real-time requirements. Second, the model’s adaptability and generalization capabilities need to be further researched for complex construction site scenarios and different types of workers. In addition, issues such as privacy protection and data security deserve attention.

Future research could focus on improving the scalability and stability of the model for application in a broader range of construction site environments. Also, exploring integrated research that combines other sensor data (e.g., sound and temperature) and artificial intelligence techniques for construction site safety monitoring is a promising direction.

## Data availability statement

The original contributions presented in the study are included in the article/**Supplementary material**, further inquiries can be directed to the corresponding author.

## Ethics statement

Written informed consent was obtained from the individual(s) for the publication of any potentially identifiable images or data included in this article.

## Author contributions

HC: conceptualization, methodology, software, investigation, formal analysis, and writing—original draft. YL: data curation and writing—original draft. HW: visualization and investigation. XH: resources and supervision. All authors contributed to the article and approved the submitted version.

## Conflict of interest

The authors declare that the research was conducted in the absence of any commercial or financial relationships that could be construed as a potential conflict of interest.

## Publisher’s note

All claims expressed in this article are solely those of the authors and do not necessarily represent those of their affiliated organizations, or those of the publisher, the editors and the reviewers. Any product that may be evaluated in this article, or claim that may be made by its manufacturer, is not guaranteed or endorsed by the publisher.
