# Supplementary material for: YOLOv5s-gnConv: detecting personal protective equipment for workers at height
Source: Front Public Health. 2023 Sep 28;11:1225478. doi: 10.3389/fpubh.2023.1225478 (PMC10569216; doi:10.3389/fpubh.2023.1225478)
Supplement: Supplementary file 1 [file Table_1.DOCX]

Supplementary Material

Article Title

Huihua Chen, Yaoyu Li*, Huanxi Wen

*** Correspondence:** Xiaodong Hu: huxiaodong1116@csu.edu.cn

# Supplementary Data

The collected dataset of workers at height totaled 10,000.


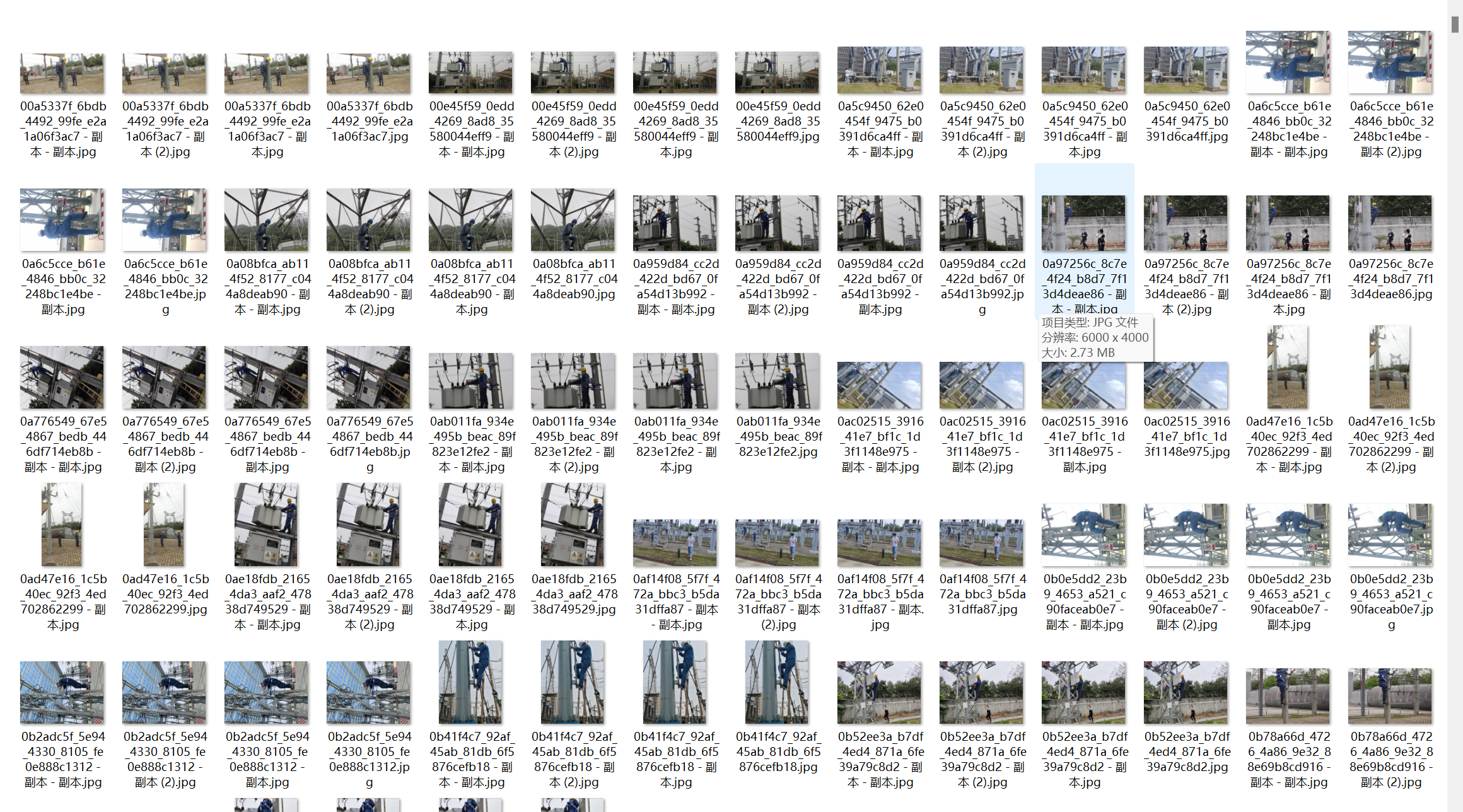


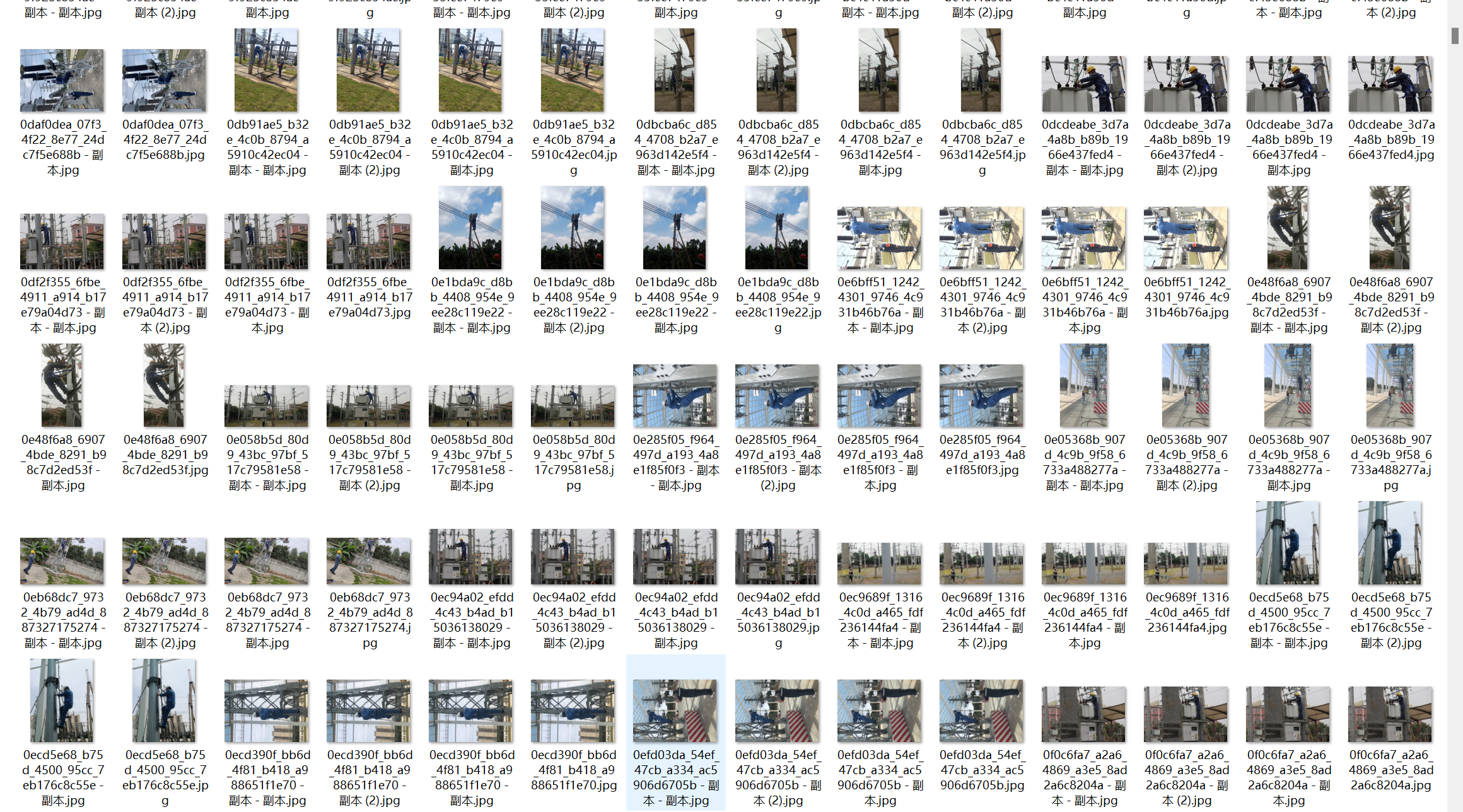


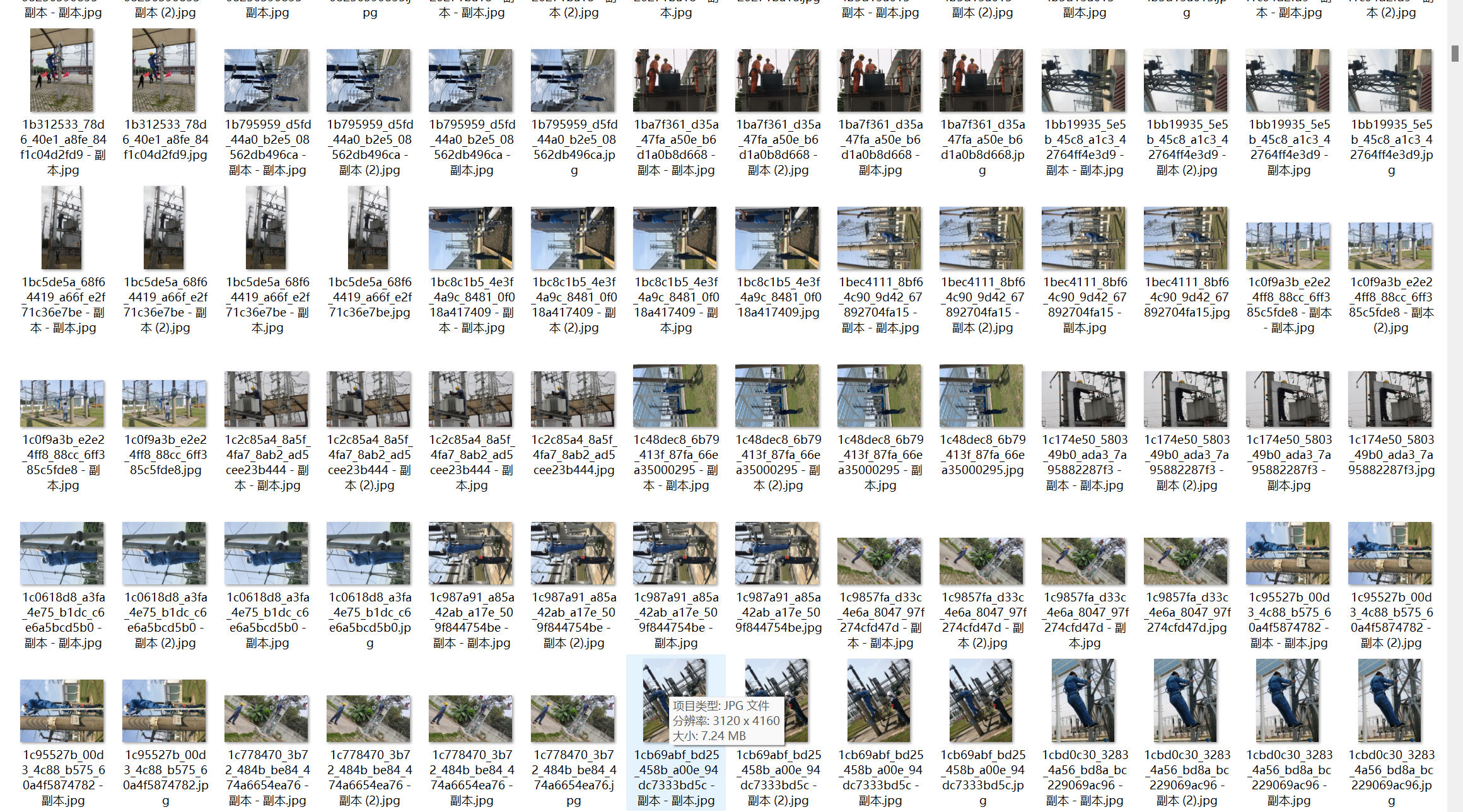


The labels of the completed dataset are shown in Table 1.

| Number of labels | Original data | Brightness | Rotation | Blur | Mosaic | Total |
| --- | --- | --- | --- | --- | --- | --- |
| people | 13966 | 27932 | 41898 | 13966 | 13966 | 125694 |
| helmet | 12857 | 25714 | 38571 | 12857 | 12857 | 115713 |
| safebelt | 11681 | 23362 | 35043 | 11681 | 11681 | 105129 |

# Supplementary Figures and Tables

## Supplementary Figures


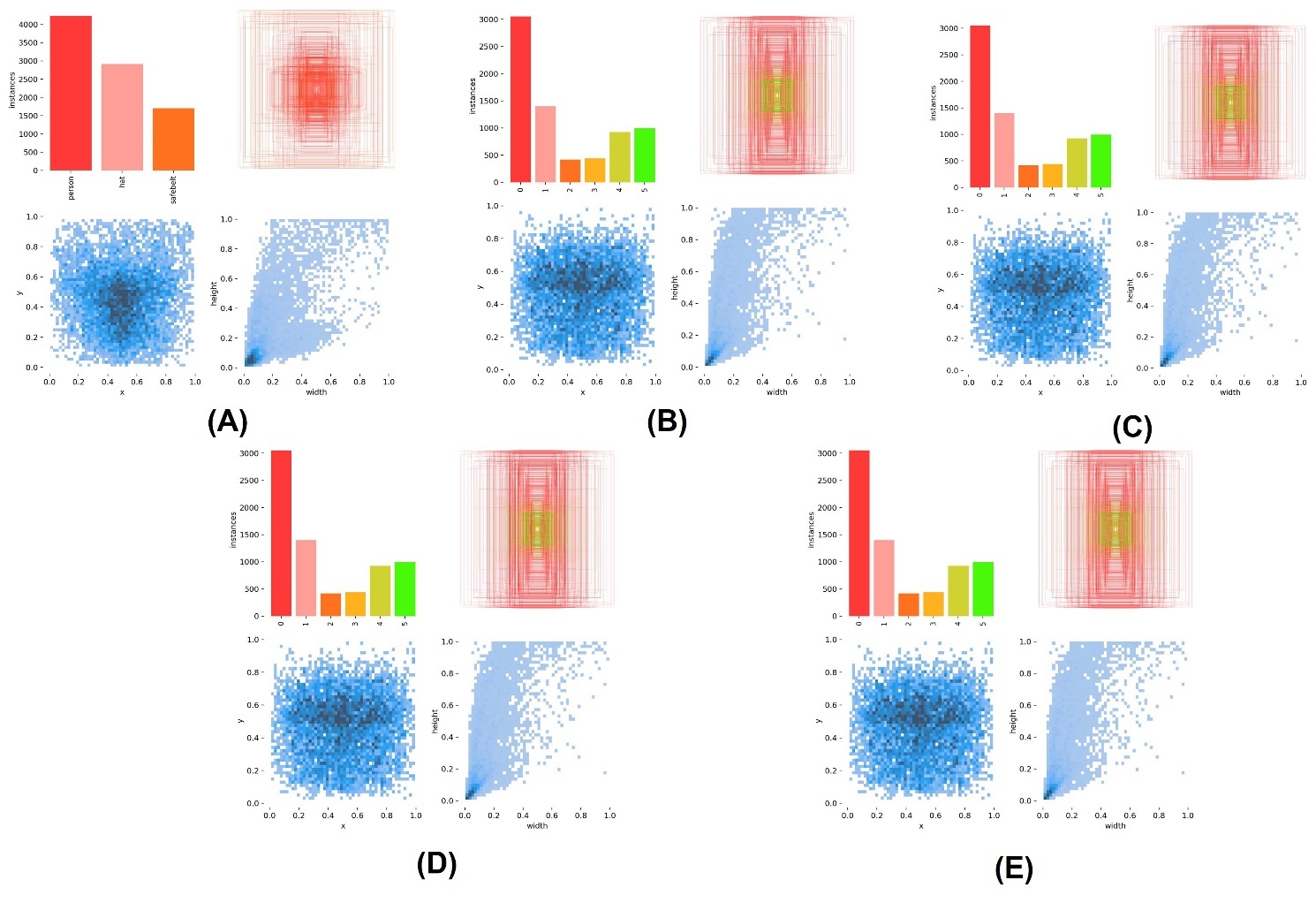


**Supplementary Figure 1.** **Label of the five models:** **(A)YOLOv5s-g^n^Conv,(B)YOLOv5s-HorBlock ,(C)YOLOv5s-HorNet,** **(D)Faster R-CNN and YOLOv5s.**


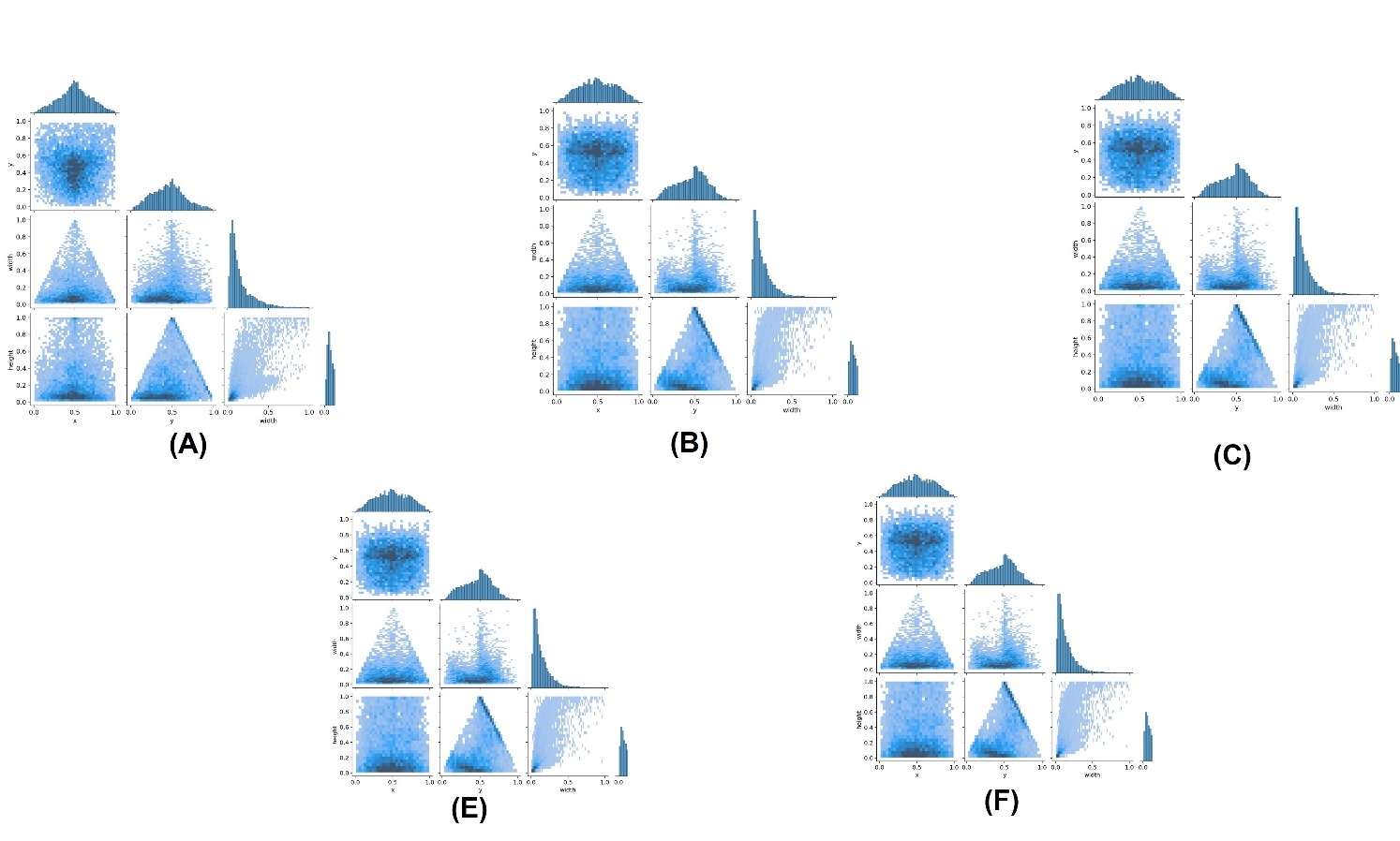


**Supplementary Figure 2.** **Label correlogram of the five models:** **(A)YOLOv5s-g^n^Conv,(B)YOLOv5s-HorBlock ,(C)YOLOv5s-HorNet,** **(D)Faster R-CNN and YOLOv5s.**

**
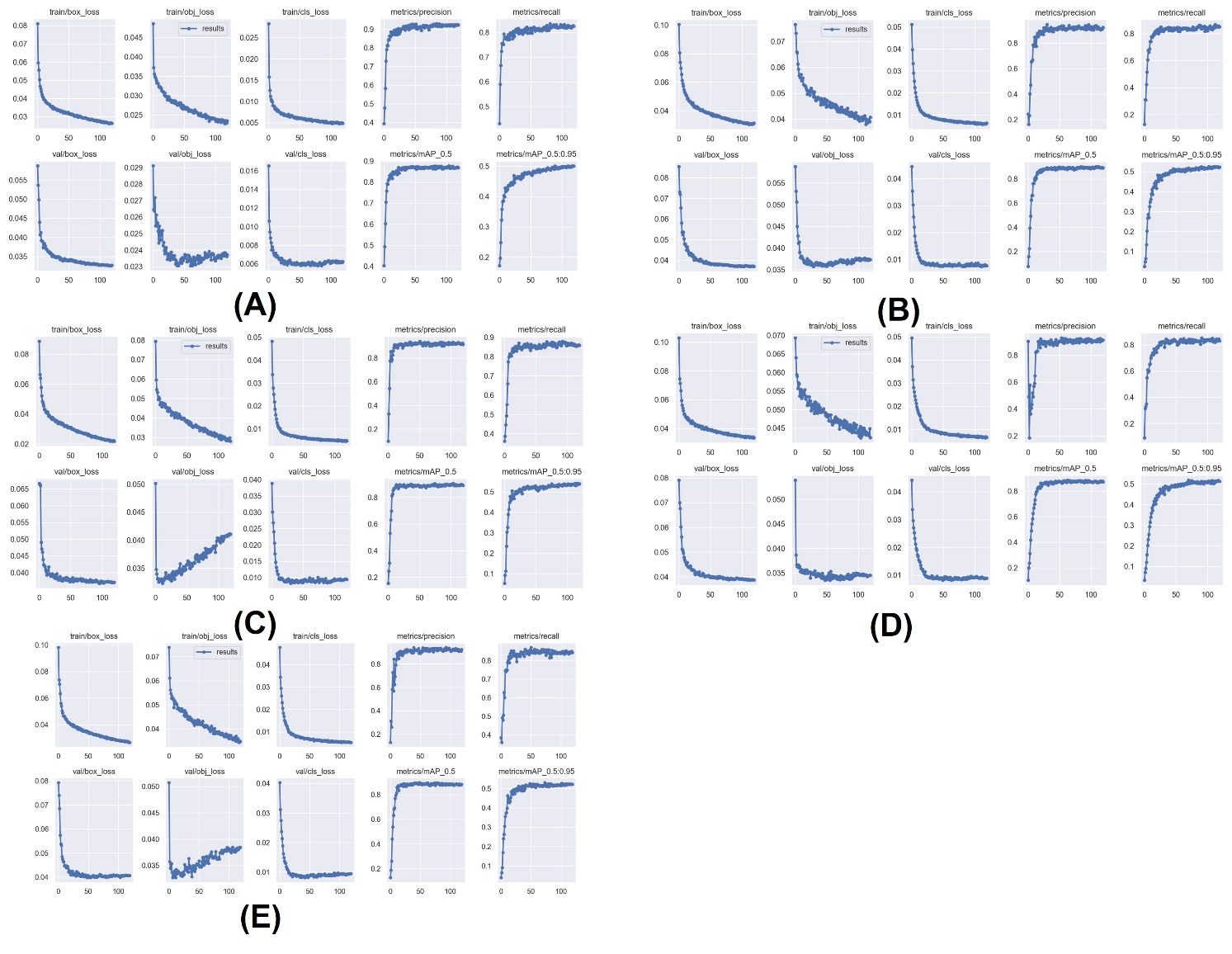
**

**Supplementary Figure 3.** **Results of the five models:** **(A)YOLOv5s-g^n^Conv,(B)YOLOv5s-HorBlock ,(C)YOLOv5s-HorNet,** **(D)Faster R-CNN and YOLOv5s.**


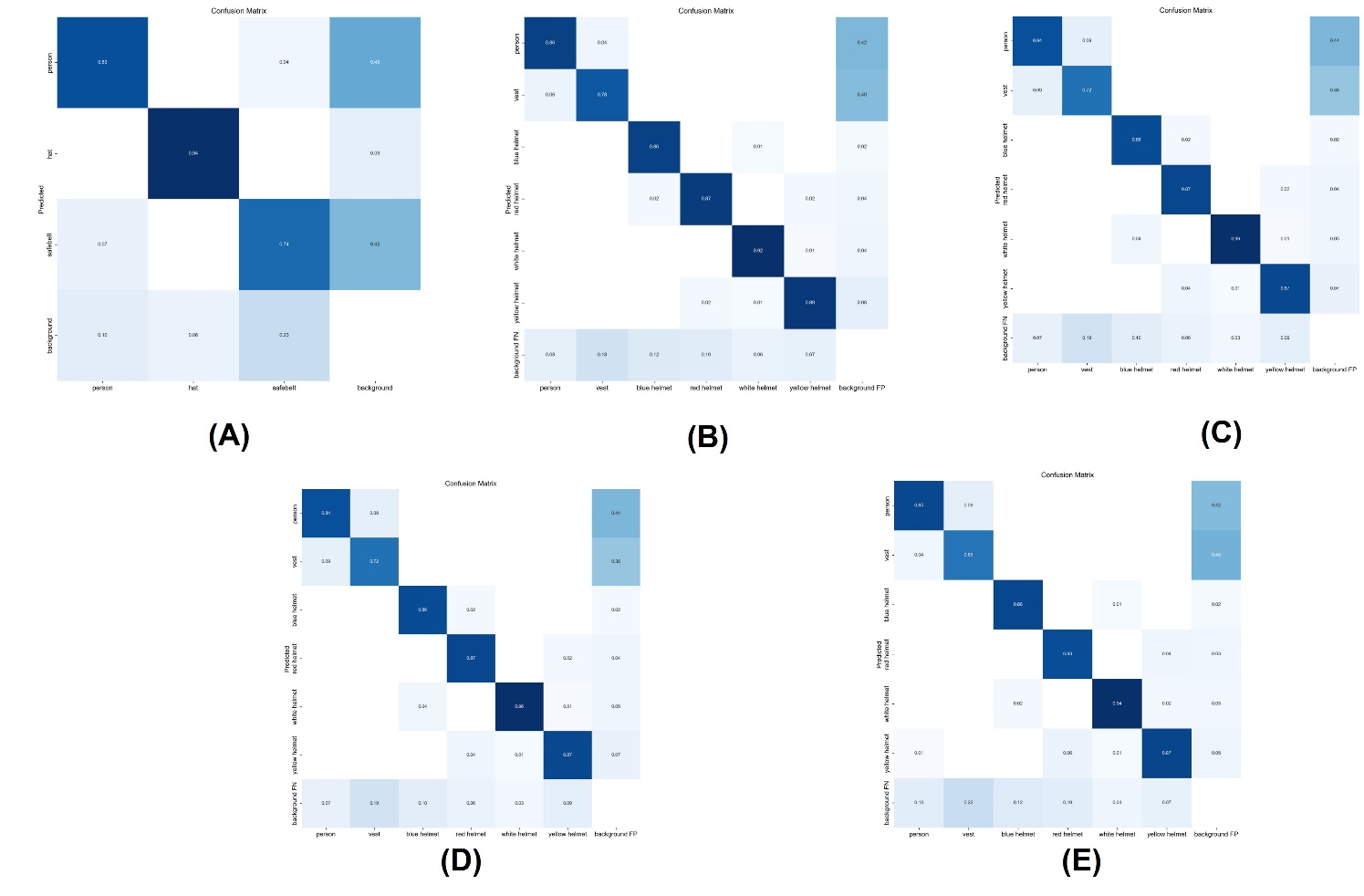


**Supplementary Figure 4.** **Confusion matrix** **of the five models:** **(A)YOLOv5s-g^n^Conv,(B)YOLOv5s-HorBlock ,(C)YOLOv5s-HorNet,** **(D)Faster R-CNN and YOLOv5s.**


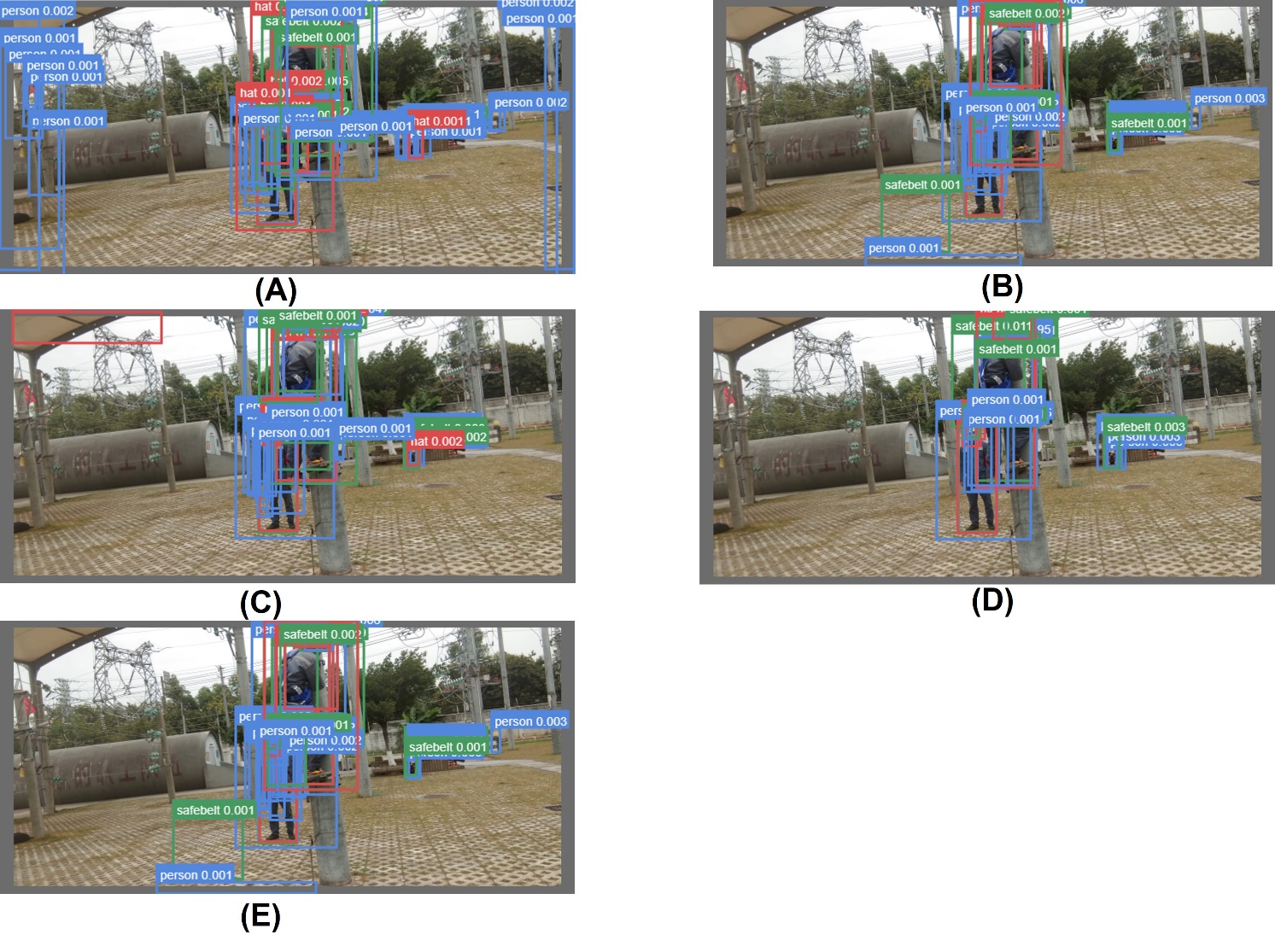


**Supplementary Figure 5.** **BoundingBoxDebugger** **of the five models:** **(A)YOLOv5s-g^n^Conv,(B)YOLOv5s-HorBlock ,(C)YOLOv5s-HorNet,** **(D)Faster R-CNN and YOLOv5s.**
